# Immune control in chronic myeloid leukemia

**DOI:** 10.18632/oncotarget.22279

**Published:** 2017-11-03

**Authors:** Mette Ilander, Satu Mustjoki

**Affiliations:** Satu Mustjoki: Hematology Research Unit Helsinki, Department of Clinical Chemistry and Hematology, University of Helsinki and Helsinki University Hospital Comprehensive Cancer Center, Helsinki, Finland

**Keywords:** chronic myeloid leukemia, NK cells, immunology, biomarker and tyrosine kinase inhibitor

Chronic myeloid leukemia (CML) is caused by the (9;22) translocation leading to the formation of the constantly active BCR-ABL fusion kinase. Tyrosine kinase inhibitors (TKIs) block the function of the oncokinase, and the use of TKIs has considerably improved the treatment responses in CML. Even though TKI treatment in CML has been a success, the long-term use may cause severe adverse effects which impact the quality of life and may cause additional morbidity. In addition, due to the high cost of the TKI therapy the lifelong treatment causes economic burden.

Several studies have shown that nearly half of the CML patients who have reached deep, durable molecular remission can stop the TKI treatment and stay in remission [1]. However, with the sensitive detection of BCL-ABL1 from the blood, it has been demonstrated that residual leukemic cells still exist in the patients who stay in remission without any treatment [2]. Although the immune system has been shown to be dysfunctional in newly diagnosed CML patients, TKI therapy may restore the function of the immune cells [3], and thus, the immune system may play a role in the leukemia control. To be able to select the best candidates for TKI stopping, it would be important to understand the biology behind a successful TKI discontinuation.

In the recent study, we evaluated in detail the phenotype and function of different immune cell subsets in CML patients who aimed to stop the TKI therapy [4]. Our results showed that at the time of TKI discontinuation patients who stayed in remission had more mature NK cells in peripheral blood expressing CD57 and CD16 antigens compared to the patients who relapsed within 6 months after imatinib stopping. In accordance, the proportion of CD56^bright^ naïve NK-cells was associated with molecular relapse. When analysing the clinical paramaters, we noticed that the proportion of NK cells was not related to CML risk score (Sokal score), previous IFN-α treatment or the duration of TKI treatment. Similar observations have also been made by other groups [5, 6]. Interestingly, we have also earlier shown that NK cells, Th1 type of response and memory T cells (both CD4+ and CD8+) are increased in CML patients who have been able to discontinue IFN-alpha monotherapy and stay in remission [7]. Similarly, in the current study where patients were treated with TKIs, the high NK cell proportion was accompanied with increased Th1 response of CD4+ T cells (TNF-α/IFN-γ production).

The classical NK cells are considered to be a part of the innate immunity. However, recent studies have demonstrated that NK cells are capable of persisting for a long time and executing memory-like functions which lead to a strong immune response when the same target is encountered the second time. These so-called adaptive NK cells have downregulated certain signalling molecules and transcription factors such as FcεRγ, PLZF, SYK, EAT-2. Interestingly, the mature NK cells in CML patients in remission after TKI discontinuation seemed to have also downregulated some of the typical adaptive NK-cell markers (such as EAT-2). In the follow-up samples after the imatinib discontinuation, we also noticed the phenotype and the amount of NK cells persisted similar as before the TKI discontinuation. Therefore, it is tempting to hypothesize that these putative adaptive-like NK cells contribute to the anti-leukemic attacks.

However, the immune system is complex and the role of other immune cell subsets is still unclear, although we could not detect clear association between other cells analysed and successful TKI discontinuation. Schutz et al. [8] have studied the dendritic cells (DC) from CML patients who discontinued TKI treatment. They demonstrated that the plasmocytoid DCs (pDCs) expressing CD86 (the ligand for CTLA4) were associated with the exhausted CD8+ T-cell phenotype and molecular relapse. This could be one of the immune escape mechanisms of CML. Based on these different studies the interaction between NK cells and pDCs should be further studied.

**Figure 1:**
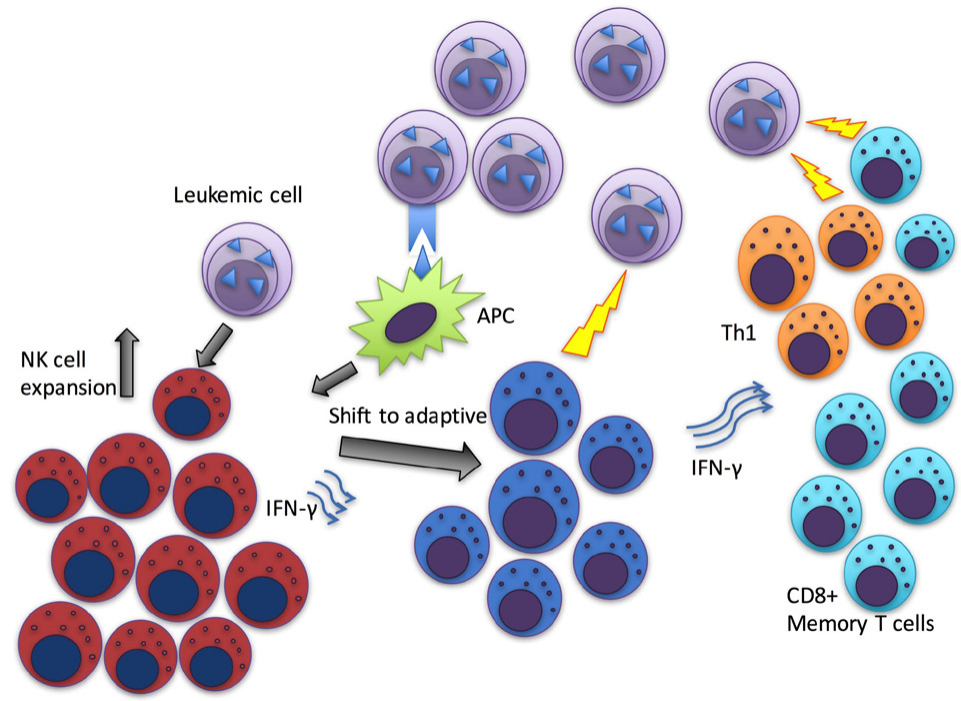
Schematic figure of the suggested hypothetical mechanism of anti-leukemia immunity in patients who have successfully discontinued TKI or IFN-α treatment Antigen presenting cells (APCs) secrete cytokines which together with the leukemia cell induced activation could induce NK cell proliferation. Repeated stimulus may shift NK cells towards more mature, adaptive phenotype. NK cells could execute either direct killing of the leukemic cells or alternatively drive T cells to a more effective leukemia targeting state.

Taken together, these results suggest that the putative anti-leukemia immunity observed as increased amount of NK cells could function through various mechanisms including both adaptive (modulation of T-cell responses) and innate immunity (direct killing effects) (Figure 1). Additional immune escape mechanisms employed by leukemic cells should be further studied when aiming at the curative treatment strategies in CML. NK cell modulating agents are already in clinical trials in other haematological malignancies and their testing in CML is warranted. These approaches could result in increased proportion of patients who can discontinue TKI treatment and therefore be closer to the cure.
